# Adeno-Associated Virus Technologies and Methods for Targeted Neuronal Manipulation

**DOI:** 10.3389/fnana.2019.00093

**Published:** 2019-11-26

**Authors:** Leila Haery, Benjamin E. Deverman, Katherine S. Matho, Ali Cetin, Kenton Woodard, Connie Cepko, Karen I. Guerin, Meghan A. Rego, Ina Ersing, Susanna M. Bachle, Joanne Kamens, Melina Fan

**Affiliations:** ^1^Addgene, Watertown, MA, United States; ^2^Stanley Center for Psychiatric Research, Broad Institute of MIT and Harvard, Cambridge, MA, United States; ^3^Cold Spring Harbor Laboratory, Cold Spring Harbor, NY, United States; ^4^Allen Institute for Brain Science, Seattle, WA, United States; ^5^Penn Vector Core, Gene Therapy Program, Perelman School of Medicine, University of Pennsylvania, Philadelphia, PA, United States; ^6^Department of Genetics, Harvard Medical School, Howard Hughes Medical Institute, Boston, MA, United States; ^7^Department of Ophthalmology, Harvard Medical School, Howard Hughes Medical Institute, Boston, MA, United States

**Keywords:** AAV, neuroscience, viral vectors, cell-type specificity, gene delivery, intersectional methods, targeted neuronal manipulation, virus technologies

## Abstract

Cell-type-specific expression of molecular tools and sensors is critical to construct circuit diagrams and to investigate the activity and function of neurons within the nervous system. Strategies for targeted manipulation include combinations of classical genetic tools such as Cre/loxP and Flp/FRT, use of cis-regulatory elements, targeted knock-in transgenic mice, and gene delivery by AAV and other viral vectors. The combination of these complex technologies with the goal of precise neuronal targeting is a challenge in the lab. This report will discuss the theoretical and practical aspects of combining current technologies and establish best practices for achieving targeted manipulation of specific cell types. Novel applications and tools, as well as areas for development, will be envisioned and discussed.

## Introduction

Understanding neural networks as they relate to development, behavior, and learning is a critical objective of neuroscience. These questions can be addressed, in part, by understanding the role of specific neural cells and brain regions, as well as the impact of individual molecules in these circuits. The successful execution of these neurobiology studies requires methods that are highly targetable, efficient, and precise. In this regard, recombinant adeno-associated viral vectors (herein referred to as AAV) are powerful tools that can be used both to target and manipulate specific neuronal subtypes (defined based on gene expression, location, and connectivity) and non-neuronal cell types within the nervous system.

**Figure 1:**
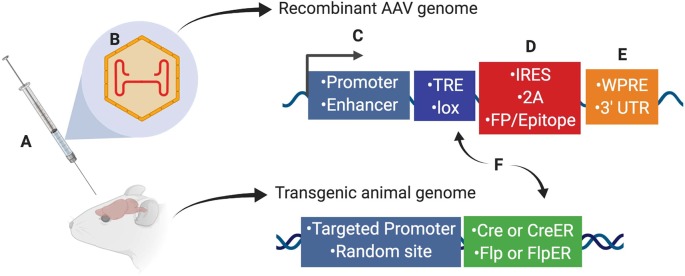
Several aspects of experimental design affect neuronal targeting and manipulation including **(A)** viral delivery method, **(B)** composition of viral capsid proteins, **(C)** promoters and/or enhancers driving transgene expression, **(D)** IRES or 2A elements for multicistronic expression coupled with fluorescent proteins (FP) or protein epitopes, **(E)** post-translational regulatory elements such as WPRE or 3′-UTR, and **(F)** Recombinase (Cre, CreER, Flp, or FlpER) expression from transgenic driver lines (inserted genomically *via* targeted or random integration) and ligand-dependent or recombinase-dependent expression elements such as TRE or lox sites, respectively. Abbreviations: TRE, tetracycline-response element; lox, LoxP sequence; IRES, internal ribosomal entry site; 2A, 2A sequence for self-cleavage; FP, fluorescent protein; WPRE, woodchuck hepatitis virus posttranscriptional regulatory element; 3′-UTR, 3′-untranslated sequence.

**Figure 2:**
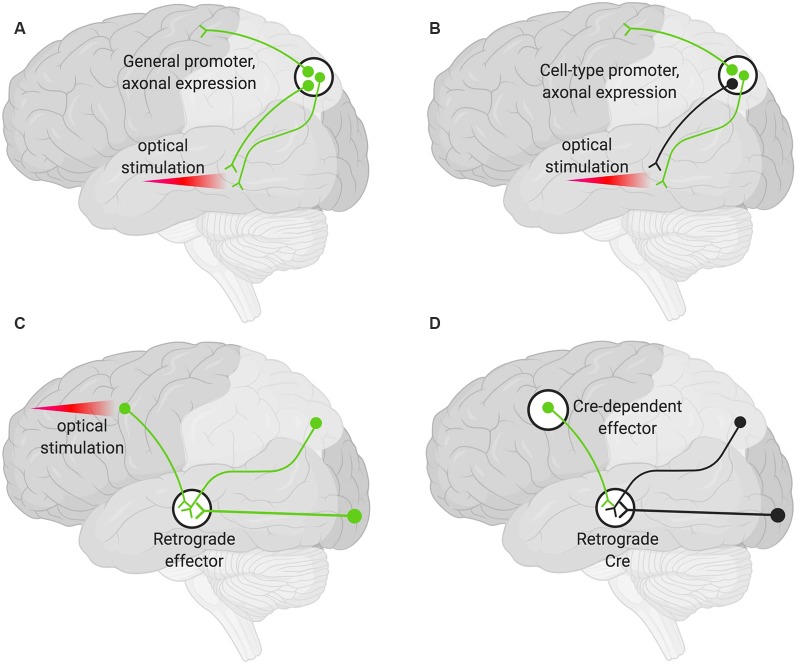
Various strategies for neuronal targeting using AAV. Delivery of neuronal effectors *via* AAV labels (green) axons and terminals with cell bodies at the injection site. **(A)** Effectors under a general promoter express in all transduced neurons with cell bodies at the injection site. Specific regions can be optically stimulated (red beam). **(B)** Effectors under cell-type promoters express only within a cell type. **(C)** Effectors delivered *via* a retrograde AAV express in all transduced neurons with axons that project into the injection site. Cell bodies in regions of interest can be optogenetically stimulated (red beam). **(D)** Delivery of a retrograde AAV expressing Cre recombinase (Retrograde Cre) to the projection site coupled with local delivery of a Cre-dependent effector limits expression to neurons within specific circuits.

Scientists using AAV for gene transfer and/or neuronal targeting must consider various questions about experimental design, including: (1) how to best deliver/administer AAV (Figure 1A); (2) which AAV serotype to use (Figure 1B); and (3) how to drive gene expression with gene regulatory elements (both within the AAV genome and the host animal or cell line; Figures 1C–F). These and many other factors can affect how efficiently cells of interest are targeted by AAV. Further, experimental parameters such as AAV titer and dosage can impact AAV efficiency, and these details are often omitted from experimental methods in the literature and can be expensive and timely to determine empirically for each study. Overall, designing an experiment with AAV is multifaceted and suboptimal experimental design can drastically reduce the quality of results. In this report, we will discuss practical aspects of using AAV and considerations for designing experiments.

## Selecting The Route of Administration and Capsid

AAV tropism, as dictated by AAV capsid proteins, is an important factor affecting transduction efficiency and specificity across cell types. Since the mechanism of AAV transduction is through the interaction of the AAV capsid with cell surface proteins and glycans, protein composition of the capsid (i.e., the AAV serotype) and the cell surface (i.e., based on cell type) determine transduction efficiency. Additionally, as the landscape of cell surface molecules varies across species, the efficiency of AAV may subsequently vary considerably across species and strains (Watakabe et al., 2015; El-Shamayleh et al., 2016; Hordeaux et al., 2018; Huang et al., 2019). Consequently, serotype and route of delivery should be carefully considered when designing experiments (Figures 1A,B). For an overview of the primary receptors for AAV serotypes, see Schultz and Chamberlain (2008).

### Direct Intraparenchymal Delivery

When injected directly into the brain, many of the naturally-occurring AAV capsids, which share homology ranging from 65% to 99% (Drouin and Agbandje-McKenna, 2013), have distinct but significantly overlapping tropisms and distribution characteristics. AAV1, AAV2, AAV5, AAV8, AAV9 and the engineered variant AAV-DJ are commonly used to target local populations of neurons after direct injections (Table 1).

**Table 1:** AAV administration routes for neuroscience.

	Administration route		
	Direct	Intravenous	Delivery into the CSF (IT/ICV/CM)
Advantages	• Regional expression achievable (serotype dependent; Kaplitt et al., 1994; McCown et al., 1996; Peel et al., 1997)	• CNS or PNS-wide transduction (Zincarelli et al., 2008)	• IT injection can be used to target spinal motor neurons and dorsal root ganglia (Zhang et al., 2010)
	• High levels of expression achievable (high MOI)	• Quick, non-invasive (Stoica et al., 2013)	• Neonatal ICV injections can provide widespread gene delivery to the CNS (Hammond et al., 2017)
	• Requires small volumes of virus	• Does not require surgical expertise (Stoica et al., 2013)	• May (Gray et al., 2013) or may not (Samaranch et al., 2011) allow CNS expression in the presence of neutralizing antibodies
	• Reduced off-target effects	• Lower more uniform expression (Chan et al., 2017)
		• Sparse labeling is possible (Chan et al., 2017)
Disadvantages	• Requires invasive surgery (Stoica et al., 2013)	• Higher dose and volume of virus required	• Expression is not confined to the CNS (Hinderer et al., 2014)
	• Damage to the targeted area (Mastakov et al., 2001; Carty et al., 2010)	• Greater risk of immune response (Colella et al., 2018)	• Requires moderately large volumes of virus
	• Challenging in certain deep brain structures	• Off-target effects may confound experiment	• Expression is not as uniform as it is after systemic delivery (Hinderer et al., 2014)
	• Transduction gradient from injection site
Expression considerations	• High levels of expression may be important for opsin expression (Yazdan-Shahmorad et al., 2018)	• Moderate expression provided by IV AAV-PHP.B/eB may be preferable for GCaMP6 expression (no nuclear expression observed), see Hillier et al. (2017)	• Expression is higher around CSF spaces and the brain/SC surface (Hinderer et al., 2014; Lukashchuk et al., 2016)
	• High-level expression makes cell-type specific transgene expression using regulatory elements more challenging
Capsids	• AAV2—confined spread, mostly neuronal (Kaplitt et al., 1994; McCown et al., 1996; During et al., 1998; Mandel et al., 1998; Davidson et al., 2000; Burger et al., 2004)	• AAV9 and rh.10—efficient neonatal CNS transduction (Foust et al., 2009, 2010; Zhang et al., 2011; Ruzo et al., 2012)	• AAV7, AAV9, and rh.10 are the most widely tested serotypes for delivery into the CSF (Federici et al., 2012; Samaranch et al., 2013; Gurda et al., 2016; Borel et al., 2016)
	• AAV-DJ—Confined spread, higher expression (vs. AAV2; Grimm et al., 2008)	• AAV-BR1—brain endothelial cell-specific (Marchiò et al., 2016)	• AAV4 enables transduction of ependymal cells (Liu et al., 2005)
	• AAV1, 5 and 8—widespread, moderate expression, neurons and glia (Burger et al., 2004; Tenenbaum et al., 2004; Cearley and Wolfe, 2006; Li et al., 2006; Taymans et al., 2007; Hadaczek et al., 2009; Dodiya et al., 2010; Masamizu et al., 2010, 2011)	• AAV-PHP.B—enhanced neuron and glial transduction after adult IV injection in mice (Deverman et al., 2016; Chan et al., 2017)	• AAV SCH9 and AAV4.18 enable SVZ progenitor cell transduction (Murlidharan et al., 2015; Ojala et al., 2018)
	• AAV2-Retro—widespread distribution, enhanced axonal uptake and retrograde expression (Tervo et al., 2016)	• AAV-PHP.eB—further evolved AAV-PHP.B variant with improved neuronal transduction (Deverman et al., 2016; Chan et al., 2017)
	• AAV1—paired with Cre exhibits trans-synaptic (anterograde) transduction (Zingg et al., 2017)	• AAV-PHP.S—evolved capsid with improved transduction of peripheral nerves and heart (Chan et al., 2017)
	• AAV2-HBKO—robust and widespread expression, primarily in neuronal cells, higher expression than parental AAV2 (Sullivan et al., 2018)
	• AAV-TT—widespread and high transduction of both glia and neuronal cells relative to parental AAV2. Wider spread than AAV9 and rh.10 (Tordo et al., 2018)

In addition to exhibiting local transduction, several serotypes exhibit transduction distal to the injection site (Burger et al., 2004; Cearley and Wolfe, 2006, 2007; Klein et al., 2006, 2008; Li et al., 2006; Reimsnider et al., 2007; Sondhi et al., 2007; Taymans et al., 2007; Cearley et al., 2008; Hollis et al., 2008; Hadaczek et al., 2009; Masamizu et al., 2011; Bu et al., 2012) and the mechanisms of these phenotypes are active areas of investigation (Castle et al., 2014). The AAV vector purification method has also been shown to impact transduction patterns (Klein et al., 2008).

Furthermore, there are important differences in how far different capsid variants spread from the injection site—AAV2 and AAV-DJ diffusion are more confined and, therefore, these capsids are often chosen for applications that require precise targeting. While expression from AAV2 is mostly neuronal, several serotypes, including AAV1, AAV5, AAV8 and AAV9, also transduce astrocytes and oligodendrocytes.

Despite the tremendous volume of work on serotype-dependent expression patterns and the complexity of the mechanisms both hypothesized and shown to drive these phenotypes, the ability to predict confidently the expression pattern in a particular experimental setup still requires empirical evidence. A non-exhaustive list of reported characteristics for several serotypes is outlined (Table 2) and can be used to narrow down suitable serotypes, though the importance of empirical validation at the onset of each study cannot be understated. Importantly, results reported in this table may vary based on anatomical region, though the results have not been summarized in this report to that degree.

**Table 2:** Transduction characteristics of select AAV serotypes.

Serotype	Transport phenotypes^a^	Transduction levels	Spread from injection site^b^	Transduced cells	Additional notes
AAV1	Retrograde (Burger et al., 2004; Reimsnider et al., 2007; Hollis et al., 2008; Bu et al., 2012)	High, similar to AAV9, AAVrh10 (Cearley et al., 2008; Aschauer et al., 2013)	Greater than AAV2, similar to AAV5, AAV8 (Burger et al., 2004)	Primarily neurons (Burger et al., 2004; Dodiya et al., 2010; Masamizu et al., 2010)	Expression levels were stable over a 9-month period at the injection site (Reimsnider et al., 2007)
	Anterograde (Cearley et al., 2008) Anterograde transsynaptic at high titers (Zingg et al., 2017)		Far from the injection site (Burger et al., 2004; Cearley and Wolfe, 2006; Sondhi et al., 2007; Taymans et al., 2007; Bu et al., 2012; Watakabe et al., 2015)	Astrocytes at low frequency (Tenenbaum et al., 2004; Li et al., 2006; Taymans et al., 2007; Hadaczek et al., 2009)	Expression *via* retrograde transduction decreased over a 9 month period (Reimsnider et al., 2007)
AAV2	Anterograde (Salegio et al., 2013)	Lower than AAV1 and AAV5 (Davidson et al., 2000; Burger et al., 2004; Aschauer et al., 2013)	Smaller than AAV1, AAV5, AAV8 and AAV9 (Burger et al., 2004; Taymans et al., 2007; Sondhi et al., 2007; Watakabe et al., 2015)	Neurons (to different degrees and not all types; Kaplitt et al., 1994; McCown et al., 1996; During et al., 1998; Mandel et al., 1998)	Expression levels were stable over a 9-month period at the injection site (Reimsnider et al., 2007)
	Retrograde at >2 months following gene transfer (Kaspar et al., 2002; Halbert et al., 2006; Sondhi et al., 2007)			Other cell types at low efficiencies (Kaplitt et al., 1994; McCown et al., 1996; Peel et al., 1997; Klein et al., 1998; Lo et al., 1999; Davidson et al., 2000; Cucchiarini et al., 2003)
				Astrocytes at low frequency (Taymans et al., 2007)	
AAV5	Anterograde (Aschauer et al., 2013)	Higher than AAV2, similar to AAV8 (Davidson et al., 2000; Taymans et al., 2007; Aschauer et al., 2013)	Greater than AAV2, similar to AAV1, AAV8, AAV9 at high doses (Burger et al., 2004; Sondhi et al., 2007; Taymans et al., 2007; Aschauer et al., 2013; Watakabe et al., 2015)	Primarily neurons (Burger et al., 2004)	Expression levels increased over time in cells at the injection site (Reimsnider et al., 2007)
	Retrograde (Burger et al., 2004; Reimsnider et al., 2007)			Astrocyte at low frequency (Tenenbaum et al., 2004; Taymans et al., 2007)	Expression *via* retrograde transduction decreased over a 9-month period (Reimsnider et al., 2007)
			Greater than AAV8 at low doses (Taymans et al., 2007)	Oligodendrocytes (von Jonquieres et al., 2013)	
AAV8	Anterograde (Masamizu et al., 2011)	Higher than AAV2, similar to AAV1, AAV5 (Taymans et al., 2007; Aschauer et al., 2013)	Greater than AAV2, similar to AAV1, AAV5, AAV9 at high doses (Sondhi et al., 2007; Watakabe et al., 2015)	Primarily neurons (Cearley and Wolfe, 2006; Masamizu et al., 2010)	Expression levels then remained stable over a 9-month period (Reimsnider et al., 2007)
	Retrograde (Masamizu et al., 2011)	Higher than AAV9 (Klein et al., 2008)	Smaller than AAV5 at low doses (Taymans et al., 2007)	Astrocytes at low frequency (Taymans et al., 2007)	Expression *via* retrograde transduction increased then decreased over a 9-month period (Reimsnider et al., 2007)
				Oligodendrocytes at low frequency (Masamizu et al., 2011; von Jonquieres et al., 2013)	
AAV9	Anterograde (Cearley et al., 2008; Masamizu et al., 2011; Castle et al., 2014)	High, similar to AAV1, AAVrh10 (Cearley and Wolfe, 2006; Cearley et al., 2008; Aschauer et al., 2013)	Similar to AAV1, AAV5, AAV8 and greater than AAV2 (Watakabe et al., 2015)	Primarily neurons (Cearley and Wolfe, 2006; Masamizu et al., 2011)	Transport and Contralateral transduction observed (Cearley and Wolfe, 2006)
	Retrograde (Cearley and Wolfe, 2006; Masamizu et al., 2011)	Lower than AAV8, similar to AAVrh10 (Klein et al., 2008)		Astrocytes (Hammond et al., 2017)
	Anterograde transsynaptic at high titers (Zingg et al., 2017)			Oligodendrocytes at low frequency (Masamizu et al., 2011)
AAV rh10	Anterograde (Klein et al., 2008)	High, similar to AAV1, AAV9, AAVrh10 (Cearley and Wolfe, 2006; Klein et al., 2008)	Far from the injection site (Burger et al., 2004; Cearley and Wolfe, 2006; Sondhi et al., 2007; Bu et al., 2012)	Primarily neurons (Cearley and Wolfe, 2006; Sondhi et al., 2007; Cearley et al., 2008)
	Retrograde (Klein et al., 2008)				

*^a^For relative retrograde transport efficiencies, see Tervo et al. (2016). ^b^For relative retrograde transport efficiencies, see Tervo et al. (2016)*.

### Systemic Delivery

Several natural AAV capsids cross the blood-brain barrier (BBB). In contrast to direct injections, intravenous injections of AAV can provide a central nervous system (CNS)-wide gene delivery. This activity is present across several species and is most pronounced when AAV is administered to the neonate. Neonatal injections of AAV9 and rh.10 have been used to transduce neurons broadly across the CNS. However, when delivered at later developmental stages including in the adult, transduction is more limited and primarily restricted to endothelial cells and astrocytes, with transduction occurring in 1–2% of neurons in the forebrain (Foust et al., 2009; Dufour et al., 2014; Deverman et al., 2016). In this context, engineered AAV capsids have provided new and dramatically more efficient options for widespread gene delivery to the CNS. The first of these vectors, AAV-PHP.B, enabled researchers to deliver genes to more than 50% of neurons and astrocytes across numerous brain regions with a single non-invasive injection (Deverman et al., 2016). Achieving this efficiency requires relatively high viral doses (~1 × 10^14^ vector genomes/kg), thus requiring large volumes of high titer virus. A further-evolved AAV-PHP.B variant, AAV-PHP.eB, addresses this issue and can achieve >50% transduction of most neuron and astrocyte populations even with a 20-fold reduction in dose (Chan et al., 2017). While the activity of the PHP capsids is not universally observed across all species, or even strains of a given species, the receptor engaged by the PHP capsids during AAV transduction has been identified and can be used to predict permissivities of cell or tissue types to these engineered capsids (Hordeaux et al., 2019; Huang et al., 2019). In addition, the same group has developed an additional AAV variant (the PHP.S variant) that can efficiently transduce dorsal root ganglia and other peripheral neuron populations following systemic administration, which should enable the study of these otherwise difficult to target peripheral neuron populations (Chan et al., 2017).

### CSF Delivery

The third option for gene delivery to the CNS is to inject vectors into the cerebral spinal fluid (CSF). Several access points can be used: the lateral ventricle (intracerebroventricular, ICV), the cisterna magna (CM), subpial (Miyanohara et al., 2016) or the intrathecal (IT) space along the spinal cord. When performed in neonates, ICV AAV administration can provide widespread gene delivery. In the adult, ICV and CM injections result in gene delivery in multiple brain regions, however, the expression is not uniform across all brain regions and superficial structures are preferentially targeted. Beyond neurons, ICV injections also provide access to periventricular cell populations. For example, after ICV injection, AAV4 can be used to transduce the ependymal cells (Liu et al., 2005), and two engineered AAV capsids, SCH9 and AAV4.18, enable transduction of subventricular zone neural progenitors (Murlidharan et al., 2015; Ojala et al., 2018). IT injection can be used to deliver genes to spinal cord motor neurons and dorsal root ganglions (Foust et al., 2010; Federici et al., 2012; Schuster et al., 2014).

### Retrograde and Anterograde Transport for Circuit Studies

AAV vectors are commonly used as part of circuit studies. Numerous natural AAV serotypes exhibit retrograde trafficking activity from their uptake at axon terminals (see Table 2). However, retrograde transduction with natural serotypes such as AAV1, AAV2, AAV6, and AAV9 requires high vector doses due to the relative inefficiency of this transduction mechanism. More recently Tervo et al. (2016) and Davidsson et al. (2018) have created modified capsids AAV2-Retro and AAV MNM008, respectively, that provide efficient transduction of neurons that send axon projections into the injection site. Transduction efficiencies of both capsids are shown to be circuit-dependent, and thus capsids should be validated for circuits of interest when planning experiments. Zingg et al. (2017) report that AAV1 and AAV9 exhibit transsynaptic anterograde transport at high titers, specifically showing that AAV1-directed Cre can activate Cre-dependent transgene expression in a post-synaptic neuron. Importantly, they note that retrograde transmission also can occur, making interpretation clear only for circuits where there is a unidirectional pattern of connectivity.

### AAV Can Cause Toxicity at High Doses

Although AAV is less inflammatory than some other viruses, it is not inert with respect to the innate (Rogers et al., 2011) or adaptive (Mingozzi and High, 2011) immune system, and may also perturb other cellular activities. Several studies have found neurotoxicity when the virus was delivered systemically or *via* direct injections into the CNS, or into the sub-retinal space of the retina. For rabies monosynaptic tracing studies, AAV is often delivered as a helper virus to supply TVA and rabies G protein. A recent study found that high doses of helper did not enable rabies infection or tracing, while diluted preparations did (Lavin et al., 2019). Although the mechanism was not determined, the authors suggest that the high doses were toxic. Similarly, neurotoxicity was seen in piglets injected systemically (Hinderer et al., 2014). In the retina, toxicity was associated with dose, if the viral promoter was expressed in a support cell type, the retinal pigmented epithelium, but not if the viral promoter activity was restricted to photoreceptor cells (Xiong et al., 2019). Innate immunity has been shown to result from stimulation of TLR9, a sensor of unmethylated CpG’s, in studies of AAV infection of muscle (Zhu et al., 2009) and liver (Martino et al., 2011). Inclusion of a short TLR9 blocking oligonucleotide within the AAV genome has been shown to alleviate this problem in some instances (Chan et al., 2019). It is thus worth carefully considering this aspect of virus dose when setting up an experiment.

In summary, a consensus of opinion has not been reached regarding the best serotype for each cell type, brain region or application. Choosing the optimal serotype requires reviewing the literature most relevant to the planned experiment and performing pilot testing for new or at least for challenging applications. As new engineered capsids with unique features continue to be developed, the available options will become more numerous and more powerful (Deverman et al., 2016; Tervo et al., 2016; Chan et al., 2017; Davidsson et al., 2018; Ojala et al., 2018).

## Controlling Gene Expression With Regulatory Elements

Cre and Flp recombinase-dependent expression elements within AAV vectors remain the go-to system for restricting transgene expression to genetically defined cell types in model organisms. However, few Cre or Flp transgenic lines have been developed in other mammalian species. Furthermore, breeding multiple transgenic lines to generate the desired offspring can be time consuming and expensive. Therefore, there is significant interest in developing the means to achieve similar expression specificity in nontransgenic animals using flexible vector-based approaches that will translate across species.

*Cis*-regulatory elements can be used to control transgene expression from AAV genomes. These elements include promoters and enhancers (Figure 1C), as well as introns, micro-RNA recognition sequences, and internal ribosome entry sites (IRES; Figure 1D) that can be used to tailor RNA processing, stability, and translation to the experimental needs. Here we will discuss how these regulatory elements can be used to restrict AAV-mediated gene expression.

### Enhancers and Promoters

Enhancer and promoters (hereafter referred to as promoters for simplicity) can generally be divided into two classes: general/ubiquitous and cell type-specific. Typically, ubiquitous promoters provide high-level, long-term expression in most cell types, though some, such as the viral CMV promoter, have been shown to exhibit silencing in specific tissues over time (McCown et al., 1996; Klein et al., 1998; Paterna et al., 2000; Tenenbaum et al., 2004; Table 3).

**Table 3:** Ubiquitous enhancers and promoters.

Promoter	Characteristics	Length (bp)	Notes	References
CMV, Cytomegalovirus early enhancer and promoter	Ubiquitous	590–800	Robust, rapid, long term expression in many cell types. Prone to silencing in some tissues, specifically the hippocampus, striatum, and substantia nigra. Silenced by 10 weeks in the spinal cord. Only modest expression in glial cells in rat. Minimal expression in rAAV2-retro helper- packaged AAV	Thomsen et al. (1984), McCown et al. (1996), Klein et al. (1998), Paterna et al. (2000), Tenenbaum et al. (2004), Gray et al. (2011) and Yaguchi et al. (2013)
CAG, CMV enhancer, CBA promoter, globin intron	Ubiquitous	1,700	Expression in excitatory and inhibitory neurons and glia	Miyazaki et al. (1989) and Nathanson et al. (2009b)
CAGGS, CMV immediate-early enhancer, CBA promoter, hybrid intron (CBA exon1/intron1/rabbit b-globin acceptor)	Ubiquitous, strong in neurons	1,600	Ubiquitous and long term expression in the brain	Niwa et al. (1991) and Klein et al. (1998)
CBh, CBA hybrid intron: CMV early enhancer, CBA promoter, CBA/MVM intron	Ubiquitous, strong in neurons	800	Stronger expression than the CBA promoter	Gray et al. (2011)
EF1a, Elongation Factor 1a	Ubiquitous, strong in neurons	1,200, 2,500	Moderate, lower expression in glia compared with CMV/CAG	Kim et al. (1990) and Gill et al. (2001)
EFS, EF1a short version	Ubiquitous	250		Montiel-Equihua et al. (2012)
UBC, Ubiquitin C	Ubiquitous, weak	400, 1,200		Seita et al. (2019)
PGK, phosphoglycerate kinase	Ubiquitous	425	Weak expression	Qin et al. (2010)

AAV and other single-stranded DNA viruses evolutionarily exhibit some of the smallest viral genomes (Campillo-Balderas et al., 2015), which has generally provided a selective pressure toward shorter promoter sequences. In contrast, the regulatory elements that control mammalian gene transcription are often distributed over thousands to hundreds of thousands of bases. Due to the limited packaging capacity of the AAV genome, identifying AAV-compatible promoters has been challenging and the development of shortened promoters is an area of active study (Nathanson et al., 2009a; de Leeuw et al., 2016). A list of cell type-specific promoters compatible with AAV vectors is provided in Table 4. High expression levels are not optimal for every application and alternative regulatory elements, such as those from the mammalian MeCP2 or PGK genes (Table 3) may be suitable for experiments where high-level viral enhancer driven expression is not desired. General recommendations for expression levels of various types of transgenes are summarized in Table 5.

**Table 4:** Ubiquitous enhancers and promoters.

Promoter	Characteristics	Length (bp)	Notes	References
hSyn1, human Synapsin1	Neuronal, broad	485	Broadly neuronal in mice, low-level expression in Purkinje cells. Excitatory neuron expression in monkeys and rats. Inhibitory neuron expression also observed, with serotype and brain-region dependent biases	Hoesche et al. (1993), Kügler et al. (2003), Dittgen et al. (2004), Nathanson et al. (2009b) and Yaguchi et al. (2013)
MeCP2, mMeCP2 promoter	Mostly neuronal, broad, weak expression	229	Expresses in neurons and in spinal cord motor neurons	Gray et al. (2011)
NSE, Neuron-specific enolase	Neuronal, broad	1,300, 1,800	Provides strong and long-term expression	Xu et al. (2001)
BM88, Neuron-specific protein	Preferentially neuronal	88		Pignataro et al. (2017)
CaMKII, Ca^2+^/Calmodulin-dependent kinase II	Neuronal, glutamatergic (cortical)	400, 1,200, 2,300	Excitatory neuron preference expression in monkeys and rat. Some inhibitory neuron expression in mouse varies with serotype, titer, and brain region	Dittgen et al. (2004), Hioki et al. (2007), Nathanson et al. (2009b), Yaguchi et al. (2013) and Scheyltjens et al. (2015)
mDLX, mouse DLX5/6 enhancer, minimal promoter and chimeric intron	Forebrain GABAergic neurons	850	Validated GABAergic neuron specificity in multiple serotypes	Dimidschstein et al. (2016)
mTH/rTH, mouse/rat Tyrosine Hydroxylase	Catecholamine neurons	2,500		Oh et al. (2009) and Chan et al. (2017)
DBH, Dopamine β hydroxylase	Adrenergic and noradrenergic neurons	1,150		Hwang et al. (2001)
PRSx8, DBH synthetic	Adrenergic and noradrenergic neurons	NR	Evaluated in noradrenergic neurons in the LC	Hwang et al. (2001)
PCP2, Purkinje Cell Protein 2 (Ple155)	Purkinje neurons	1,650		de Leeuw et al. (2016) and Chan et al. (2017)
FEV, ETS transcription factor (Ple67)	Serotonergic neurons	2,000	Serotonergic neurons	de Leeuw et al. (2016) and Chan et al. (2017)
MCH, Melanin-concentrating hormone	Subpopulation, dorsal lateral hypothalamus	830		van den Pol et al. (2004)
SLC6A4, Serotonin Transporter (Ple198)		3,050	Expression is strongest in the thalamus	de Leeuw et al. (2016)
NR2E1 (Ple264)	Müller glia	2,030		de Leeuw et al. (2016)
GfABC1D, truncated GFAP	Astrocytes	680		Lee et al. (2008)
Aldh1l1	Astrocytes	1,300		Koh et al. (2017)
mMBP, mouse myelin basic protein	Oligodendrocytes	1,900		Gow et al. (1992)
MAG, Myelin-Associated Glycoprotein	Oligodendrocytes	300, 1,500, 2,200	All provide expression in oligodendrocytes, 1,500 and 2,200 bp versions are more specific	von Jonquieres et al. (2016)
ICAM-2, Intracellular Adhesion Molecule 2	Endothelial	330		Cowan et al. (1998)
CLDN5, Claudin5 (Ple261)	Endothelial	2,960		de Leeuw et al. (2016)
Tie-2, TEK Receptor Tyrosine Kinase	Endothelial	730		Leung et al. (2009)
vWF, von Willebrand Factor	Endothelial	730		Jahroudi and Lynch (1994)
FLT1, Endothelial Growth Factor Receptor	Endothelial	1,030		Morishita et al. (1995)
TRE, rtTA-tTA responsive element	Inducible	320–400, (1,400 w/tTA)		Chenuaud et al. (2004) and Chan et al. (2017)
c-FOS	Activity-dependent	760		Ye et al. (2016)
eSARE	Activity-dependent	980		Kawashima et al. (2014)

**Table 5:** General expression considerations for specific transgenes and applications.

Transgene	Application	Optimal expression level
Opsins	Optogenetics	High
DREADDs	Chemogenetics	Low for optimal specificity
Ca^2+^ sensors	Activity monitoring	Moderate
Voltage sensors	Activity monitoring	Moderate-high
dLight1	Dopamine indicator	Not yet known
Fluorescent reporters (XFPs)	Expression reporters, protein tagging	Moderate
Luciferase/AkaLuc	Expression reporter	Low-High
Cre/FlpO/Dre/KD/B3Recombinases	Intersectional expression/Circuit studies	Low
CRISPR-Cas9	Gene editing	Moderate-High
TVA and rabies G	Circuit studies/retrograde tracing/TRIO/cTRIO	TVA (low) Rabies G (Moderate)

### Multicistronic Vectors

Although AAV vectors have a limited packaging capacity, it is possible to express multiple short transgenes from a single vector using one of several approaches: (1) separate translational units where each cDNA is controlled by separate 5′ and 3′ regulatory elements; (2) using IRES sequences to insert two separate translational units into a single mRNA; or (3) the use of viral 2A sequences to generate separate proteins from the same translational unit (Figure 1D). Table 6 highlights considerations for choosing between the use of IRES sequences and 2A “self-cleaving” peptides.

**Table 6:** Bicistronic expression options.

	Bicistronic expression elements	
	2A	IRES
Advantages	• Requires minimal sequence space	• Protein products are unmodified
	• Results in similar expression of both proteins
	• Can be used to express >2 proteins
Disadvantages	• May reduce expression of both proteins	• May not provide equivalent expression of both transgenes
	• Adds peptide sequence to the C-terminus of the first protein and proline to the N-terminus of the second protein	• May not provide equivalent expression of both transgenes
	• Digestion at the 2A site is not always complete and may lead to fusion protein expression	• IRES sequences are >500 bp

One important consideration when evaluating expression strategies and determining specificity is that the expression levels required for reporter detection may not match what is necessary for the activity of an opsin, DREADD or recombinase. For example, fluorescent proteins are commonly used to evaluate gene regulatory elements and vector design. However, fluorescent reporter assays may give the false impression of specificity if high levels of expression are seen in target cell types and low-level expression goes undetected in off-target populations. If these same regulatory elements are then used to drive DREADDs or Cre, which can mediate their effects at low expression levels, then the specificity may appear reduced. If this goes unexamined, then the interpretation of experimental results could be compromised. Moreover, though they are commonly used, fluorescent proteins are not necessarily inert and can lead to immune responses in larger animals (Samaranch et al., 2014), and over-expression related toxicities in mice.

### Post-transcriptional Regulatory Elements

Transgene expression can also be controlled post-transcriptionally through the use of elements impacting RNA splicing, nuclear export, stability, and translation into proteins (Figure 1E). Inclusion of an intron can have positive impacts on expression levels. Introns have also been combined creatively with recombinase sites and partially inverted transgenes to achieve tight intersectional control of transgene expression (Fenno et al., 2014, 2017). Many recombinant AAV genomes also include a woodchuck hepatitis virus posttranscriptional regulatory element (WPRE), which can dramatically enhance expression. For several examples of how the inclusion of a WPRE affects expression from AAV vectors please see de Leeuw et al. (2016).

Complementary miRNA target sites (TS) are frequently engineered into the 3′-untranslated region (3′-UTR) of recombinant AAV vector genomes to mitigate off-target transgene expression. These sequences are complementary to miRNAs expressed within off-target cell types but not within the target population. miRNA binding to the perfectly complementary miRNA TS results in degradation of the RNA. Inclusion of multiple copies of the short miRNA TS sequences can dramatically lower off-target transgene expression. For example, by incorporating three copies each of miR-1 and miR-122, which are specifically expressed in muscle and liver, respectively, Xie et al. (2011) reduced transgene expression from intravenously administered AAV9 in muscle, heart, and liver, while maintaining brain expression. miRNA TS that enhance the restriction of lentiviral mediated gene expression to GABAergic neurons have also been identified (Xie et al., 2011; Keaveney et al., 2018), as have miRNA TS that result in more selective GCaMP6f expression from a strong ubiquitous promoter (Challis et al., 2019). Given their short lengths, miRNA TS can be multiplexed within the same genome, making them attractive elements for reducing expression outside of the cell type of interest.

### Conditional Expression

The mammalian CNS contains extremely diverse types of neurons. These neuronal cell types can be distinguished by their intrinsic gene expression profiles, which are differentially regulated throughout development. Binary expression systems of drivers and reporters can be used to drive transgene expression in genetically-defined cells (reviewed in Huang et al., 2014).

There are two general strategies for using binary expression systems to access genetically-defined cell types: transactivation-based systems (based on tetracycline (TET)-response elements, TRE) and recombination-based systems (based on lox-site recombination, lox) described in (Taniguchi, 2014; Figure 1F). Gene targeting techniques can be used to insert *Cre* or *Flp* site-specific recombinases in the mouse genome. These knock-in driver mouse lines express Cre or Flp under the activity of a target gene’s endogenous promoter. Thus, Cre and Flp driver mouse lines constitute a genetic switch to turn on a recombinase-dependent reporter or effector. Since the recombinase is only expressed in cells defined by the target gene’s endogenous promoter activity, this system allows labeling and manipulation of neurons defined by the targeted gene’s expression pattern. A recently developed strategy can direct Cre or Flp activity to cells expressing GFP (Tang et al., 2017). Nanobodies directed against GFP have been engineered as fusion proteins with Cre or Flp. The recombinases are active only in GFP-expressing cells. This method can be used for the activity of only one recombinase, or can be used intersectionally, e.g., mating GFP and Cre mouse lines, infecting with a GFP-dependent Flp-nanobody fusion, and then using a Cre+ and Flp-dependent readout (see further below for intersectional methods). For temporal control, one can use TET-inducible systems to provide rapid and detectable expression at a given time point (Hioki et al., 2009; Sadakane et al., 2015). The TET system also has the advantage of amplified gene expression (Watakabe et al., 2012; Chan et al., 2017). This may be particularly important in studies on non-human primates since transduced cells could be maintained long-term without affecting cell integrity (Sadakane et al., 2015).

The reporter or effector whose expression is dependent on the driver’s activity is introduced *in vivo* by either crossing the driver to a transgenic reporter mouse line, using a viral vector, or electroporating the DNA construct into the cells. With the widely used Cre or Flp drivers, the conditional reporter expression depends on the recombination of specific lox or frt sites, respectively. In the case of the TET system, a tet-on, reverse tetracycline transactivator (rtTA) or tet-off (tTA) driver is paired with an AAV vector with a promoter harboring tet-responsive elements (TRE).

#### Targeting at Random vs. Targeting to a Specific Gene’s Locus

Knock-in Cre and Flp recombinases or GFP can be inserted into the genome either randomly or at a particular gene locus (Figure 1F). Conventional transgenic and BAC transgenic approaches result in relatively random insertions into the genome. However, knock-in mouse lines targeted to a specific locus by homologous recombination have the advantage that the expression of the inserted gene will recapitulate the expression pattern within cells of the endogenous gene of interest. There are several advantages to using targeted knock-in driver mouse lines (Table 7). Targeted knock-in of Cre or Flp or GFP into a specific gene’s transcription/translation initiation site can result in the recapitulation of expression of the endogenous locus. When using this strategy, it should be noted that individuals can exhibit variable levels of silencing. Typically, an optimal “non-silencing” male should be identified and used for genetic crosses. However, the offspring of this male may exhibit silencing and must be revalidated.

**Table 7:** Methods of delivering Cre for cell-type targeting, labeling, and manipulation.

	Method of Cre delivery		
	Targeted knock-in Cre mouse line	Transgenic Cre mouse line (not generated by homologous recombination)	Cre *via* AAV
Advantages	• Specific and reliable by genetic targeting to the locus of interest (higher certainty that driver activity will reflect the endogenous expression of the gene of interest)	• Cheap and easy to produce mouse lines	• Stable over time
	• Comprehensive with Cre mouse lines	• Lower Cre expression than AAV	• Spatial control: can restrict delivery to a particular region
	• Sparse if using CreER by adjusting tamoxifen dose	• Can be delivered broadly by systemic (e.g., tail vein or retro-orbital) injection
	• Can combine with viral strategies to achieve spatial control or very strong expression
	• Lower Cre expression than AAV
Disadvantages	• Time-consuming and costly to produce and maintain mouse lines	• Does not necessarily recapitulate the endogenous gene’s expression	• Expression gradients from injection site(s)
	• Genetic silencing in mouse lines can affect Cre expression	• AAV vectors increase interleukin levels in the animal	
		• Sexual dimorphism can arise that does not reflect the gene’s native expression profile	• High levels of Cre protein exhibit cell toxicity
		• Transgenic animals can lose specificity over time	

#### Temporal Control With Tamoxifen

To control the expression of a reporter in a subset of neurons that uniquely and transiently express a certain marker at a particular time point, tamoxifen-inducible recombinases (Feil et al., 2009), so-called CreER or FlpER, can be used as drivers. In CreER driver mice, activation of the expressed Cre recombinase requires administration of the estrogen receptor modulator drug tamoxifen to the animal, allowing for temporal control of recombination.

The typical tamoxifen dose varies from 10 to 200 mg/kg, depending on the desired degree of recombination at the reporter allele. In effect, this controls the sparseness/density of the labeling of the targeted neuronal population. The timing for tamoxifen administration depends on the temporal characteristics of the promoter driving CreER in the specific population to be targeted. Importantly, the half-life of tamoxifen (approximately 48 h) must be taken into consideration. Tamoxifen preparation is detailed in the published protocol in (Vaughan et al., 2015).

Tamoxifen can be administered in one of three ways, depending on the desired developmental time point for activating the CreER driver: oral gavage to the mother for embryonic induction; subcutaneous injection to offspring for early postnatal induction, or intraperitoneal injection in offspring for late postnatal and adult induction.

#### Best Practices for Induction of CreER or FlpER Mouse Lines

To generally improve the reliability of results obtained with CreER or FlpER mouse lines, several tamoxifen doses should be evaluated. If the level of recombination of the reporter achieved with a high dose of tamoxifen is low, administration of 4-hydroxytamoxifen (the native form of tamoxifen) can improve activation of CreER (Jahn et al., 2018). Tamoxifen needs to be metabolized in the liver to reach its active 4-hydroxytamoxifen form. Tamoxifen is often preferred over 4-hydroxytamoxifen for routine applications in highly active CreER driver lines due to lower cost and improved solubility. Following tamoxifen induction, CreER or FlpER will be active for ~48 h, which may impact induction during developmentally active time periods. 4-hydroxytamoxifen is a better choice for tighter control of the window of activation. Administration of tamoxifen or 4-hydroxytamoxifen by gavage to the pregnant mother to induce the pups at embryonic timepoints can lead to problems of miscarriage or poor mothering. When administering tamoxifen at embryonic time-points, use of Swiss or CD1 compared to C57Bl6 mice can improve outcomes in two ways: they produce larger litters and females are better mothers, which overall can improve pup survival.

#### Validating a Knock-in Driver Mouse Line

Knock-in driver mouse lines are designed to control the expression of reporter probes, sensors, and effectors in genetically-defined cell-types. It is important to validate that the driver mouse line expressing a site-specific recombinase (e.g., Cre, CreER, Flp, FlpER) reflects the endogenous expression pattern of the gene targeted by the knock-in driver. Various approaches can be used separately or jointly to validate a knock-in driver mouse line: (1) Crossing the mice with a suitable reporter like Rosa26-CAG-LSL-td-tomato (Ai14) or Rosa26-CAG-LSL-h2B-GFP and assessing brain-wide expression; (2) Immunostaining of the target regions; and (3) dual fluorescent *in situ* hybridization (dual fISH) with probe for reporter (e.g., RFP or GFP for Rosa26-CAG-LSL-td-tomato (Ai14) or Rosa26-CAG-LSL-h2B-GFP, respectively). Note that assessing Cre lines by crossing to a reporter line gives an integrated view of Cre activity over the lifetime of the animal. To assess Cre activity in the target cell population at the particular age of interest, a viral vector with a Cre-dependent reporter in a Cre line can be used. Similarly, for CreER lines tamoxifen induction should be performed at different developmental time-points to assess temporal specificity.

In addition, leakiness of both mouse lines and viruses must be assessed prior to the interpretation of experimental results that rely on the complete restriction of transgene expression. Leakiness of a mouse line can be assessed when validating the mouse line, ensuring that expression of the site-specific recombinase or activator (i.e., Cre, CreER, Flp, FlpER or tTA) is consistent with the gene of interest in its native form. Crossing the driver to a reporter (e.g., fluorescent reporter) and performing dual fluorescent *in situ* hybridization (dual fISH), with probes for the fluorescent reporter and for the gene of interest allows one to check that endogenous expression of the gene and activity of the driver are present in the same cells. Using knock-in driver mouse lines instead of randomly inserted BAC or plasmid driver mouse lines allows for targeted expression, with a higher likelihood of expression within cells expressing the endogenous gene of interest. Leakiness of the tamoxifen-inducible driver mouse line can be assessed by crossing the inducible driver CreER or FlpER to a Cre or Flp dependent fluorescent reporter and checking for expression of the fluorescent protein without administering tamoxifen. Finally, leakiness of the Cre- or Flp-dependent or tTA-activated AAV can be checked by injecting the virus in a mouse that has been crossed to a fluorescent reporter mouse line for the driver/activator. If expression from the virus and from the mouse reporter line match, this indicates that the AAV is specific to the driver/activator. One further evaluation of a given virus prep for background recombination (or leak) is to inject it into a mouse line that does not encode the cognate recombinase. Recombination can occur during the growth of the AAV plasmid in bacteria, and/or during virus preparation in mammalian cells, and should be assessed using this test.

## Viral Strategies for Targeting Defined Populations

Combining various experimental techniques can enable the precise targeting of specific neuronal populations of interest. The general advantage of using combinatorial approaches is that the specificity of cell targeting can be improved with each additional technique. Applying known characteristics of AAV and the genetically encoded transgenes they carry, neuronal populations can be specifically targeted and manipulated based on their locations and connectivity. Here we will review several targeting strategies.

### Axon Terminals

A viral encoded transgene can be targeted to axon terminals. Such terminals can then be targeted optogenetically in specific areas by only delivering light to the region harboring the terminals of interest (Figure 2A). This technique can be further restricted such that only axons of a particular cell type are targeted by using cell-type-specific promoters to drive the AAV expression (Figure 2B). Additional specificity can be achieved by choosing an injection site, AAV serotype, and titer so that only desired cell types are infected (Nassi et al., 2015).

### Projection Neuron Targeting

Targeting the cell bodies of projection neurons can be important for manipulating only those neurons that terminate at a particular site. To target these neurons, an AAV capable of retrograde transduction (e.g., AAV2-Retro) can be delivered to projection sites and the cell bodies of those neurons terminating at that region will be targeted (Tervo et al., 2016). This method alone will not give rise to pathway specificity. However, if used to deliver optogenetic tools, light can be delivered only to the region harboring the cell bodies of interest (Figure 2C; Nassi et al., 2015).

Finally, specific populations of projection neurons can be targeted by coupling local delivery of a Cre-dependent, inducible neuromodulator (e.g., a DREADD or opsin) with retrograde delivery of Cre to the site where the targeted projection neurons originate (Figure 2D). Using this approach, a subset of projection neurons (Figure 2D), rather than all the projection neurons (Figure 2C), then express the Cre-dependent neuromodulator. This has the advantage that effectors (e.g., the DREADD ligand) can then be delivered systemically rather than locally and still only manipulate the subset of projection neurons that have been targeted (Nassi et al., 2015).

## Areas for Development

The arsenal of new sensors, actuators, recombinases, genes, RNA and base editing enzymes, and other genetically encoded tools for studying the nervous system is rapidly growing. AAV vectors remain the most versatile and powerful approach for delivering these tools to the CNS. Nevertheless, delivery challenges remain and efforts are ongoing to develop new vectors that address several key needs including: (1) improved widespread CNS gene transfer *via* IV and ICV routes; (2) AAV vectors capable of more efficient trans-synaptic anterograde transport; (3) vector solutions for delivering transgenes too large to fit in a single AAV virus; (4) capsids that specifically target defined neural cell types and neuronal subtypes; and (5) viral vectors that enable transduction of microglia.

Overall, consistency and repeatability of both existing and newly developed AAV tools can be improved by following best practices and guidelines. While powerful technologies are being developed, each has technical limits that need to be considered both when designing experiments and when interpreting results. To this end, improved platforms for sharing information, including technical guidelines and best practices, will serve the research community by enabling technologies to be used to their fullest capacities consistently across labs.

## Author Contributions

LH, KG, MR, IE, SB, JK, and MF planned and hosted the workshop where this material was discussed. BD, KM, AC, KW, and CC discussed and established the best practices for the field based on their expertise. LH, BD, and KM wrote the manuscript, which was then edited by AC, KW, CC, KG, MR, IE, SB, JK, and MF.

## Conflict of Interest

LH, KG, MR, IE, SB, JK, and MF were employed by Addgene, a nonprofit repository that distributes research materials including plasmids and AAV.

The remaining authors declare that the research was conducted in the absence of any commercial or financial relationships that could be construed as a potential conflict of interest.
